# *Lycium europaeum* Extract: A New Potential Antioxidant Source against Cisplatin-Induced Liver and Kidney Injuries in Mice

**DOI:** 10.1155/2018/1630751

**Published:** 2018-09-05

**Authors:** Ilhem Rjeibi, Anouar Feriani, Anouar Ben Saad, Jazia Sdayria, Issam Saidi, Sana Ncib, Sami Souid, Mohamed Salah Allagui, Najla Hfaiedh

**Affiliations:** ^1^Research Unit of Macromolecular Biochemistry and Genetic, Faculty of Sciences of Gafsa, 2112 Gafsa, Tunisia; ^2^Common Services Unit for Research, Faculty of Sciences of Gafsa, 2112 Gafsa, Tunisia; ^3^Laboratory of Animal Ecophysiology, Faculty of Science of Sfax, 3018 Sfax, Tunisia

## Abstract

This study was designed to assess the protective effects of *Lycium europaeum* methanol extract (LEM) on liver and kidney injuries induced by cisplatin. The phytochemical composition, the antioxidant activity, and hepatorenal injury biomarkers were investigated. Results revealed that LEM exhibited a significant antioxidant activity *in vitro* on DPPH radical and H_2_O_2_ scavenging assays. In the animal studies, treatment with LEM significantly reduced the effects of cisplatin intoxication on serum liver biomarkers and serum renal biomarkers. Meanwhile, LEM diminishes significantly the effect of cisplatin on the level of lipid peroxidation in liver and kidney tissues. The activities of the antioxidant enzymes (reduced glutathione, glutathione peroxidase, superoxide dismutase, and catalase) were increased in groups pretreated with LEM and quercetin. Additionally, the normal histological structures of the liver and kidney were restored after treatment with LEM. This work clearly demonstrated that *L. europaeum* may be useful as a drug with hepato-nephroprotective potentials.

## 1. Introduction

*Lycium*, a member of the Solanaceae family, consists of 70 species distributed throughout temperate and subtropical areas. *Lycium* was cited in the traditional medicine of many countries. In Tunisia, the dry fruits from *L. intricatum* are traditionally used to protect from eye diseases [1]. In China, *L. chinense* and *L. barbarum* are used for longevity, infertility, and headache [2]. The flowers from *Lycium shawii* are used as antidiabetic [3] and leaves are used to prevent stomach ache; in addition, the infusion from roots can treat ulcer and back pain. Furthermore, several studies have reported that plants from this genus exhibited many pharmacological properties such as antiaging and neuroprotective effects [4, 5], hepatoprotective activities [6], and antitumor and immunomodulation activities [7]. Phytochemical investigation of this genus demonstrated that polysaccharides are extensively distributed in fruits [8, 9]. Moreover, a number of works showed that *Lycium* is a rich source of phenolic acids, carotenoids, tannins, flavonoids, mineral, and vitamin [9, 10]. *L. europaeum* is a shrub spread in many Mediterranean regions [11, 12]. In folk medicine, the aerial part of this plant is used for the treatment of inflamed skin. Few studies of *L. europaeum* have been conducted on the fruits. Findings obtained from 80% ethanol extract demonstrated the cytoprotective effects against human lung carcinoma and rat adrenal medulla cancer cells [13]. Such activities were essentially related to free radical scavenging capacities, as that has been demonstrated in many works [13, 14]. In addition, fruit extracts from *L. europaeum* are characterized by high content of flavonoids, anthocyanins, carotenoids, lycopene, and tannin [13]; these constituents have indicated potential biological activities. In our previous studies, we have demonstrated that *L. europaeum* leaf extracts have antinociceptive and protective effects against CCl_4_-induced liver and kidney injuries in mice [15]. In this study, the phytochemical composition of the leaf of *L. europaeum* methanol extract (LEM) was evaluated. The antioxidant activity *in vitro* and the hepato-nephroprotective effects of this plant were also investigated.

## 2. Materials and Methods

### 2.1. Chemicals

Cisplatin (cis-dichlorodiammine platinum (II)), quercetin, butylated hydroxytoluene (BHT), dimethyl sulfoxide (DMSO), hydrogen peroxide (H_2_O_2_), 2-thiobarbituric acid, trichloroacetic acid, catalase (CAT), malondialdehyde (MDA), and 2,2-diphenyl-1-picrylhydrazyl (DPPH) were purchased from Sigma–Aldrich Co. (Sigma, St. Louis, USA). Standards (iron (Fe), zinc (Zn), calcium (Ca), potassium (K), magnesium (Mg), manganese (Mn), cuivre (Cu), and sodium (Na)) were obtained from Merck (Darmstadt, Germany). Superoxide dismutase (SOD) and glutathione peroxidase (GPx) assay kits were purchased from Randox Laboratory Ltd. Methanol was purchased from PanReac (Barcelona, Spain). All other chemicals and solvents were of analytical grade and were obtained from Sigma–Aldrich (Sigma, St. Louis, USA).

### 2.2. Plant Material and Preparation of Extract

The leaves of *Lycium europaeum* were sampled from Redeyef (latitude: 34° 38′ 3^″^ N, longitude: 8° 18′ 03^″^ E, and altitude: 623 m), southwestern Tunisia, in January 2016 and deposited at the herbarium in the Faculty of Sciences, University of Gafsa, Tunisia. Leaves (450 g) were washed with distilled water and dried at room temperature (approximately 20% relative humidity and 24°C temperature) for 48 h. Then the samples were ground to fine powder using a blender (Moulinex, France) and extracted for 24 h with methanol for three times at room temperature with magnetic stirring. After filtration using Whatman number 1 filter paper, the solvent was entirely evaporated under vacuum at 40°C using a Büchi rotary evaporator, to obtain the dry extracts (LEM). The phytochemical composition of LEM included caffeic acid, gallic acid, catechin, epicatechin, apigenin, coumaric acid, luteolin, naringenin, vanillic acid, rutin, quercetin, and kaempferol [15].

### 2.3. The Mineral Compositions

Minerals were evaluated according to the method described by Kachiguma et al. [16] with minor modifications, using atomic absorption spectrophotometer (AAS) (ZENIT series). Calibration curves were constructed using the standard stock solutions of 1000 mg/L which were diluted to make working standards of different concentration, and the calibration ranges were selected according to the expected concentrations of the elements of interest. The standard, blank, and sample solutions were then read on AAS at the following wavelengths: 248.2 nm for iron, 766.5 nm for potassium, 285.2 for magnesium, 257.2 nm for manganese, 317.9 nm for calcium, 327.3 nm for copper, 589.5 nm for sodium, and 213.2 nm for zinc.

### 2.4. Determination of Lipid-Soluble Pigments

Lipid-soluble pigments were estimated using the method of Arnon [17]. A dried leaf extract (100 mg) was extracted with acetone-hexane solution (4 : 6). The absorbance of the filtrate was measured at 400–700 nm using UV-Vis spectrophotometer.

The chlorophyll contents were calculated through the following formulas and then expressed as *μ*g/g of dry weight:

(1) $$\begin{array}{c} Chlorophyll\;a\;\left(Chl\;a\right)\left(mg/100\;mL\right)=0.999\;\left(A_{663}\right)-0.0989\;\left(A_{645}\right),\\ Chlorophyll\;b\;\left(Chl\;b\right)\left(mg/100\;mL\right)=0.328\;\left(A_{663}\right)-1.77\;\left(A_{645}\right),\\ \beta -Carotene\;\left(mg/100\;mL\right)=0.216\;\left(A_{663}\right)+1.220\;\left(A_{645}\right)-0.304\;\left(A_{505}\right)+0.452\;\left(A_{453}\right),\\ Lycopene\;\left(mg/100\;mL\right)=-0.0458\;\left(A_{663}\right)+0.204\;\left(A_{645}\right)-0.304\;\left(A_{505}\right)+0.452\;\left(A_{453}\right). \end{array}$$

### 2.5. Hydrogen Peroxide (H_2_O_2_) Scavenging Activity

The effect of *L. europaeum* extract on scavenging H_2_O_2_ was determined based on the protocol described by Ruch et al. [18] with some modifications. Briefly, a mixture of 1.6 mL of phosphate buffer (0.1 M, pH 7.4) and H_2_O_2_ solution (0.4 mL, 40 mM) was added to 1 mL of LEM with a different concentration (10, 20, 30, 50, 100, and 150 *μ*g/mL). The tubes were vortexed and incubated at room temperature for 10 min. BHT was used as standards for comparison. The absorbance was measured at 230 nm.

(2) $$\begin{array}{c} I\left(\%\right)=\frac{absorbance\;control-absorbance\;sample}{absorbance\;control}\times 100. \end{array}$$

### 2.6. DPPH Radical Scavenging Activity

The effect of the extract on DPPH radical was determined following the method reported by Bounatirou et al. [19]. Adequate solutions of extract (5, 10, 20, 30, 50, 80, and 100 *μ*g/mL) were prepared to obtain a final volume of 1 mL and were mixed with 2 mL of a freshly prepared DPPH solution (0.1 mM, in methanol). After 30 min of incubation in the dark, the absorbance was measured at 517 nm on a UV-VIS spectrophotometer. IC_50_ value (amount of sample providing 50% of scavenging on DPPH) was determined from the linear regression equation obtained from the concentrations of the sample and the percentage of inhibition.

### 2.7. Antioxidant Activity of LEM *In Vivo*

#### 2.7.1. Experimental Animals

Male Swiss mice, approximately 20–24 g body weight (bw), with age of about 7 weeks were obtained from the Central Pharmacy (Tunisia) and were maintained for a two-week adaptation period. Animals were fed with 15% protein food pellets (SNA, Sfax, Tunisia) and had tap water *ad libitum* and were cared according to the Tunisian code of practice for the Care and Use of Animals for Scientific Purposes and the European convention for the protection of vertebrate animals used for experimental and other scientific purposes (Council of Europe Number 123, Strasbourg, 1985).

#### 2.7.2. Treatment

The animals (*n* = 30) were divided into five groups, with six mice in each group. Hepatorenal toxicity in mice was induced by intraperitoneal injection of cisplatin dissolved in corn oil at the dose of 13 mg per kg body weight (bw) [20]. Treatment was then carried out as follows:

Group (I) *Control*. Corn oil was administered orally (p.o.) for 10 days.
Group (II) *LEM, 150 mg/kg bw* [15]. Animals were treated for 10 days with LEM dissolved in corn oil at 150 mg per kg bw doses p.o.
Group (III) *Cisplatin*. Corn oil was administered orally for 10 days and a single intraperitoneal (i.p.) dose of cisplatin (13 mg/kg, i.p.) was administered on the 5th day.
Group (IV) *LEM + cisplatin*. Mice were treated with LEM (150 mg per kg bw doses p.o.) orally for 10 days and a single dose of cisplatin (13 mg/kg, i.p.) dissolved in corn oil was administered on the 5th day, 1 h after the LEM dose.
Group (V) *Quercetin + cisplatin.* Animals were treated with quercetin dissolved in DMSO (0.5% *v*/*v*) at 50 mg per kg bw doses p.o. [21] orally for 10 days and a single dose of cisplatin (13 mg/kg, i.p.) on the 5th day, 1 h after the quercetin dose.

All animals were killed by decapitation 24 h after the last treatment. Blood samples were collected into heparinized or EDTA tubes by putting a funnel under the neck's mice after cervical decapitation to avoid stress. Blood was centrifuged at 1500 rpm for 10 min in order to obtain serum for biochemical analysis. The kidneys and livers were immediately removed and weighed for the organ weight ratio calculation.

#### 2.7.3. Serum Biochemical Analysis

Serum liver biomarkers (alanine aminotransferase (ALT), aspartate aminotransferase (AST), and lactate dehydrogenase (LDH)) were assayed using commercial reagent kits from Biomaghreb (Tunisia). Biochemical markers of kidney injury (urea, creatinine, uric acid, and blood urea nitrogen (BUN)) were estimated using commercial reagent kits (Sigma–Aldrich) according to the manufacturer's protocol.

#### 2.7.4. Tissue Homogenate Preparation

About 1 g of each organ was cut into small pieces and immersed into a 2 mL ice-cold lyses buffer (tris-buffered saline, pH 7.4) using Ultra-Turrax homogenizer for 15 min and then filtered and centrifuged (9000 rpm, 30 min, 4°C). Supernatants were stored at −80°C until use.

#### 2.7.5. Protein Quantification in the Liver and Kidneys

Protein contents in homogenates were measured using the Folin reaction through the method of Lowry et al. [22] using bovine serum albumin as the standard at 660 nm.

#### 2.7.6. Evaluation of Lipid Peroxidation Levels

To estimate the degree of lipid peroxidation, 100 *μ*L of each homogenate was added to 100 *μ*L of trichloroacetic acid (TCA, 5%) and the mixture was centrifuged at 4000 × g for 10 min. After that, 100 *μ*L of the supernatant and 200 *μ*L of thiobarbituric acid reagent (TBA, 0.67%) were incubated for 15 min on a boiling water bath. The level of lipid peroxidation was measured as thiobarbituric acid reactive substances (TBARS) and was expressed as malondialdehyde (MDA) formation, in accordance with the method of Draper and Hadley [23] and expressed in nmoles/mg of tissue.

#### 2.7.7. Antioxidant Enzyme Assays

Superoxide dismutase (SOD) activity was estimated according to the method of Durak et al. [24] and was expressed as units/mg protein. Catalase (CAT) activity was measured using the technique of Aebi [25] and was expressed as *μ*moles H_2_O_2_ destroyed/min/mg protein. Glutathione peroxidase (GPx) activity was determined using the protocol described by Flohe and Gunzler [26] and was expressed as U/mg protein.

#### 2.7.8. Histopathological Studies

Liver and kidney sections were fixed using formalin solution (10%). Then tissues were washed in 70% alcohol, dehydrated through a series of ascending ethanol percentage washes until 100% ethanol was reached, cleared in xylene, infiltrated with soft paraffin (melting point = 60°C), and finally embedded into molten paraffin. The paraffin sections were dewaxed in xylene and then rehydrated through a series of descending ethanol percentage washes finally embedded into molten paraffin wax. The resulting paraffin blocks were cut at 4–6 mm thickness using a rotatory microtome stained with hematoxylin and eosin and observed under a microscope.

### 2.8. Statistical Analysis

Statistical analysis was performed using the SPSS version 18.0 software. All data were analyzed using ANOVA followed by the Tukey test. All values are given as mean values and standard deviation. Differences were considered significant at *p* < 0.05.

## 3. Results and Discussion

### 3.1. Phytochemical Compositions and *In Vitro* Antioxidant Effect of *L. europaeum*

Minerals have significant roles in many activities in the human body. For example, the dietary potassium and calcium supplementation play an important role in the regulation of blood pressure [27, 28].

The nutritional composition of the leaf from *Lycium* and the information about the mineral contents are limited. The micromineral (Fe, Cu, Mn, and Zn) and macromineral (Ca, Mg, Na, and K) profiles of *L. europaeum* are detailed in Table 1. Results revealed that K (1528.32 mg/100 g) is the most important macronutrient in the leaf sample. According to Kim et al. [29] and Endes et al. [30], K was the concentrated mineral in the leaves of *L. chinense* and *L. barbarum.* Moreover, the results showed that the leaf contains a higher Fe level compared to the other micronutrients. All over, the mineral composition of *Lycium* is greater than or equal to other leafy vegetables [31].

With regard to the pigment contents in the extracts from the leaves of *L. europaeum*, Table 2 indicates high amounts of chlorophylls (382.19 *μ*g/g dry weight), followed by *β*-carotene (68.02 *μ*g/g dry weight), and lycopene (40.38 *μ*g/g dry weight). Lipid-soluble pigments like chlorophylls, *β*-carotene, and lycopene, play an important in the human health [32]. *β*-Carotene and chlorophyll have been known to have antioxidant properties, being capable of inhibiting lipid oxidation [33]. They showed the ability to reduce risks of chronic diseases induced by free radicals. Lycopene has been demonstrated to prevent diverse pathologies such as cancer and cardiovascular diseases [32]. The presence of valuable quantities of nutrients (dietary minerals) and lipid-soluble bioactive pigments like chlorophylls, *β*-carotene, and lycopene, makes the leaf from *L. europaeum* a special component for human nutrition.

The antioxidant assays have revealed the ability of *L. europaeum* methanol extract to scavenge the DPPH and H_2_O_2_ radicals (Table 2). The EC_50_ values for the DPPH- and H_2_O_2_-scavenging activities were 23.02 and 28.83 *μ*g/mL, respectively. This great antioxidant activity could be explained by the presence of bioactive compounds in leaves such as carotenoids and lycopene. The fruits of *L. europaeum* have been proved to be rich in antioxidant compounds (anthocyanins, carotenoids, lycopene, and tannin) [13]. Recently, Rosa et al. [12] have demonstrated a great antioxidant activity of *L. europaeum* fruit oil in the DPPH and ABTS scavenging tests. Moreover, our recent research has found that the leaves of *L. europaeum* consisting of bioactive compounds, including caffeic acid gallic acid, naringenin, and another 9 compounds, could protect against CCl_4_-induced liver and kidney injuries in mice, *in vivo*, according to HPLC-DAD analyses [15].

### 3.2. Effects of *L. europaeum* on the Body Weight and Organ Weights

Cisplatin is a chemotherapeutic drug used to treat several tumors. Unfortunately, numerous severe side effects of this drug have been reported like neurotoxicity, hepatotoxicity, and nephrotoxicity [34]. In recent times, phytochemicals from plants have shown protective effects against liver and kidney injuries induced by cisplatin administration [35, 36].

The effects of *L. europaeum*, quercetin, and cisplatin on the body weight as well as the relative kidney and liver weights were illustrated in Table 3. During the experimental period, all the studied groups survived. Although, a significant decrease in body growth and an increase in liver and kidney weights were recorded in cisplatin-treated groups, as compared to the controls. The reduction in the body weight could be attributed to a decrease in the food consumption or to the effects of this toxic drug on the gastrointestinal tract [37]. Many studies have demonstrated that cisplatin administration can induce a rise in kidney weights [35, 38]. When quercetin or *L. europaeum* was added to the treated animals, the adverse effects of cisplatin on these parameters were reversed.

### 3.3. Effects of *L. europaeum* on Serum Liver and Kidney Biomarker Levels

A number of studies have demonstrated the hepatorenal toxicity of cisplatin [35, 39]. The deleterious effect of cisplatin on liver function may be revealed by the alteration in liver biomarker enzymes including AST and ALT. In fact, the damage in hepatocytes leads to changes in their membrane permeability, resulting in the escape of enzymes from cells [40]. Likewise, the level of LDH is a strong indicator of apoptosis, since it is freed by cells in response to a condition of apoptosis [41]. In this study, the activities of AST, ALT, and LDH in serum increased significantly (*p* < 0.001) with cisplatin compared to the control group (Table 4).

To investigate the effect of cisplatin on kidney function, biomarkers of kidney injury such as urea, creatinine, uric acid, and BUN were analyzed.  Table 4 shows that the levels of urea, creatinine, uric acid, and BUN in the cisplatin-treated animals increased significantly to 68.01 *μ*mol/L, 96.01 *μ*mol/L, 30.58 *μ*mol/L, and 8.99 mmol/L as compared with the control groups at 35.51 *μ*mol/L, 52.29 *μ*mol/L, 20.39 *μ*mol/L, and 4.63 mmol/L, respectively. These results were consistent with previous reports [20, 35, 42]. The administration of *L. europaeum* methanol extract showed normalization in these renal biochemical parameters. This effect of the extract was similar to that of quercetin, the standard antioxidant compound. The hepatoprotective and renoprotective activities of *L. europaeum* were due to its antioxidant effect. The study of Domitrović et al. [20] demonstrated that cisplatin-mediated kidney injuries in mice were significantly ameliorated by berberine through the reduction of nuclear factor-kappa-*β* translocation and the inhibition of tumor necrosis factor-*α* and cyclooxygenase-2 expression. The most recent study of Sánchez-González et al. [43] showed that the nephroprotective effect of quercetin could be attributed to its antioxidant, anti-inflammatory, and vascular potentials.

### 3.4. Effects of *L. europaeum* Methanol Extract on the SOD, CAT, and GPX Levels in the Liver and Kidneys

**Figure 1:**
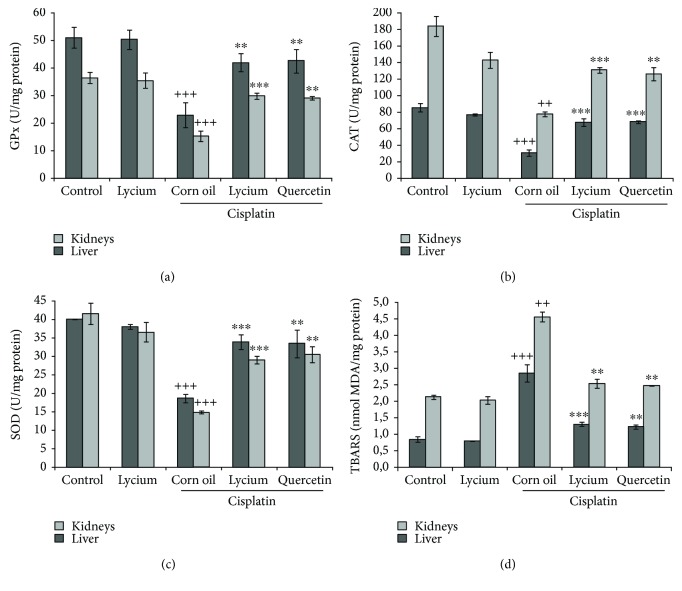
Lipid peroxidation and antioxidant enzyme activities in liver and kidney tissues of mice treated with cisplatin. Values were expressed as the mean ± SD (*n* = 6). ^++^Significant difference at *p* < 0.01 and ^+++^significant difference at *p* < 0.001: the cisplatin group versus the control group. ^∗∗^Significant difference at *p* < 0.01 and ^∗∗∗^significant difference at *p* < 0.001: the *Lycium* + cisplatin and quercetin + cisplatin groups versus the cisplatin group.

The antioxidant system of defense includes three enzymes (SOD, CAT, and GSH) which play an important role in preventing cells against reactive oxygen species and their harmful effects. However, prolonged use of chemotherapeutic drugs like cisplatin induced acute liver and kidney injuries, which make them more sensitive to oxidative damage [39]. The effects of cisplatin and LEM pretreatment on liver and kidney oxidative stress parameters are illustrated in Figure 1. It was clearly remarkable that cisplatin administration produced a statistically significant decrease (*p* < 0.001) in the levels of SOD, CAT, and GSH both in the liver and in the kidney as compared to control groups. These results are in accordance with earlier findings of Kumar et al. [35] who reported decreased activities of the above-mentioned antioxidant enzymes, in liver and kidney tissues of rats after cisplatin administration. In contrast, treatment with LEM at a dose of 7.5 mg/kg BW inhibited the above-elevated parameters significantly compared to the cisplatin-treated group (*p* < 0.001). The preventive effect of LEM was similar to that of quercetin-treated mice (50 mg/kg BW). Clearly, our results indicate that the methanol extract from *L. europaeum* prevents the exhaustion in enzymatic antioxidants and protect from cisplatin-induced oxidative damage.

### 3.5. Effects of *L. europaeum* Methanol Extract on the Lipid Peroxidation Inhibitory Activity

MDA, the final product of lipid peroxidation, is generated under overproduction of free radicals and is regarded as a marker of cell injury and cytotoxicity [44].

MDA levels in the liver and kidney are illustrated in Figure 1(d). A significant increase in the liver MDA level was found in the group receiving cisplatin (2.85 nmol/mg protein) when compared to the control group (0.85 nmol/mg protein), (*p* < 0.001). This increase in MDA levels following cisplatin exposure may be attributed to the overproduction of ROS, which could be associated with hepatocyte enzyme leakage and the inhibition of mitochondrial enzyme activity [45]. Kidney MDA levels were also markedly increased in the cisplatin treated group (4.55 nmol/mg protein), compared to the untreated control group (2.13 nmol/mg protein) (*p* < 0.01). Conversely, pretreatment with LEM prevented the increase of MDA content in liver and kidney tissues. Meanwhile, the treatment with quercetin showed a good protective effect compared to the cisplatin-treated group (*p* < 0.01).

One of the possible mechanisms of hepatic and renal protective actions of *L. europaeum* is via antiperoxidative properties and potential role in defense against free radicals.

### 3.6. Effects of *L. europaeum* Methanol Extract on the Morphology of the Liver and Kidneys

**Figure 2:**
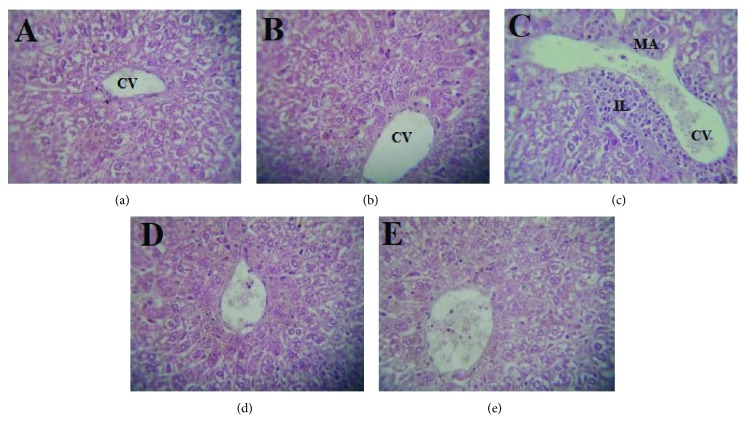
Effects of the methanol extract of leaves from *Lycium europaeum* (LEM) on the hepatic histology in CCl_4_-intoxicated mice. Livers were sectioned and stained with hematoxylin-eosin (H&E). Representative photographs (200x) from the control group (a), 150 mg/kg BW LEM group (b), cisplatin-treated group (c), 150 mg/kg BW LEM + cisplatin group (d), and 50 mg/kg BW quercetin-positive control group (e). MD: membrane cell degradation; IL: inflammatory leukocyte infiltrations; CV: central vein.

**Figure 3:**
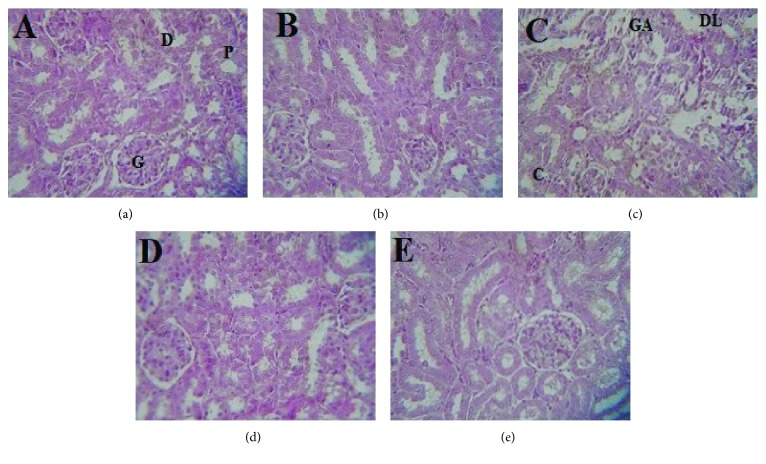
Effects of the methanol extract of leaves from *Lycium europaeum* (LEM) on kidney histology in cisplatin-intoxicated mice. Kidneys were sectioned and stained with hematoxylin-eosin (H&E). Representative photographs (200x) from the control group (a), 150 mg/kg BW LEM group (b); cisplatin-treated group (c), 150 mg/kg BW LEM + cisplatin group (d), and 50 mg/kg BW quercetin-positive control group (e). GA: glomerular atrophy; DL: tubular dilation; G: glomerule; D: distal convoluted tubules; P: proximal convoluted tubules; C: coast.

The hepatorenal protective potentials of LEM and quercetin were confirmed by histopathological examination (Figure 2). The control group (Figure 2(a)) and LEM group (Figure 2(b)) showed normal histological structure of the liver. In contrast, the cisplatin-treated group showed significant morphological changes in the liver section (Figure 2(c)). These alterations were marked by membrane cell degradation; casts are observed in some tubules, multiple foci of hemorrhage and major vascular congestion that may be awarded to the toxic effects of cisplatin by the generation of ROS, which caused damage to different membrane constituents of the hepatocytes. The administration of LEM (Figure 2(d)) and quercetin (Figure 2(e)) has suppressed the above changes and the liver section had architecture nearly comparable to that of the control group without any signs of vascular or inflammatory changes. On the other hand, the kidneys of the control group (Figure 3(a)) and LEM group (Figure 3(b)) showed normal histological structure. However, cisplatin-treated mice showed visible pathological changes, including glomerular atrophy, infiltration of inflammatory cells, and degenerated tubular structure (Figure 3(c)). These renal lesions were remarkably ameliorated by treatment with LEM and quercetin (Figures 3(d) and 3(e)). These results suggested that *L. europaeum* has a certain protective effect against cisplatin intoxication which is consistent with antioxidant enzyme and biochemical results.

## 4. Conclusion

The data from this study revealed, for the first time, the antioxidant activity and phytochemical content of the methanol extract from *Lycium europaeum.* Results indicated that LEM is rich in bioactive compounds. LEM was found to have strong hepatorenal protective effect against cisplatin-induced liver and kidney injury in mice. This beneficial effect may be related to the strong antioxidant activity and free radical-scavenging capacity of this plant. Our results proved that *L. europaeum* is a novel source of natural antioxidant and encourage its use to prevent liver and kidney dysfunctions.

## Figures and Tables

**Table 1:** Mineral content of *Lycium europaeum.*

Minerals	Amount (mg/kg)
Potassium (K)	12451.26 ± 205
Sodium (Na)	59.21 ± 2.62
Magnesium (Mg)	925.54 ± 13.20
Calcium (Ca)	1105 ± 25.54
Iron (Fe)	52.15 ± 1.03
Zinc (Zn)	10.21 ± 0.61
Copper (Cu)	10.21 ± 0.14
Manganese (Mn)	4.05 ± 0.06

Values are means ± SD of three separate experiments.

**Table 2:** Phytochemical contents and antioxidant activity of *Lycium europaeum.*

Phytochemical contents (*μ*g/g dry weight)				Scavenging activity (EC_50_, *μ*g/mL)			
				DPPH		H_2_O_2_	
Chlorophyll a	Chlorophyll b	*β*-Carotene	Lycopene	LEM	Vitamin C	LEM	BHT
329.61 ± 21.02	52.58 ± 3.12	68.02 ± 2.31	40.38 ± 1.05	23.2 ± 1.05	17.63 ± 0.81	28.83 ± 1.54	19.90 ± 0.64

Values represent mean ± SEM from three independent experiments. LEM: *Lycium europaeum* methanol extract; BHT: butylated hydroxytoluene.

**Table 3:** Effects of *Lycium europaeum*, quercetin, and cisplatin on the BW gain and liver and kidney index.

Parameters	Groups				
	Control	LEM	Cisplatin	LEM + cisplatin	Quercetin + cisplatin
Initial BW (g)	21.92 ± 0.62	22.18 ± 0.18	21.63 ± 0.37	21.99 ± 0.42	21.67 ± 0.34
Final BW (g)	27.50 ± 1.21	27.64 ± 0.61	22.59 ± 0.28^++^	26.49 ± 0.9^∗∗^	25.89 ± 0.62^∗∗^
BW Gain (%)	20.20 ± 3.02	19.73 ± 1.24	4.27 ± 0.65^++^	16.86 ± 4.12^∗∗^	16.21 ± 3.33^∗∗^
Liver weight (g)	1.22 ± 0.01	1.25 ± 0.00	1.69 ± 0.01^+++^	1.50 ± 0.01^∗∗∗^	1.43 ± 0.03^∗∗^
Kidney weights (g)	0.24 ± 0.02	0.26 ± 0.00	0.35 ± 0.02^++^	0.28 ± 0.01^∗∗^	0.28 ± 0.01^∗∗^
Liver index (%)	4.40 ± 0.14	4.52 ± 0.13	7.49 ± 0.04^+++^	5.65 ± 0.19^∗∗^	5.51 ± 0.07^∗∗∗^
Kidneys index (%)	0.87 ± 0.06	0.98 ± 0.03	1.56 ± 0.10^++^	1.07 ± 0.07^∗∗^	1.09 ± 0.08^∗∗^

Values represent mean ± SEM from three independent experiments, *n* = 6 mice per group. ^++^Significant difference at *p* < 0.01 and ^+++^significant difference at *p* < 0.001: the cisplatin group versus the control group. ^∗∗^Significant difference at *p* < 0.01 and ^∗∗∗^significant difference at *p* < 0.001: the LEM + cisplatin and quercetin + cisplatin groups versus the cisplatin group. Organ index (%) = (organ weight/body weight) × 100; LEM: *Lycium europaeum* methanol extract; BW: body weight.

**Table 4:** Effects of *Lycium europaeum* and quercetin on serum biochemical parameters of cisplatin-treated mice.

Groups	Kidney function				Liver function		
	Urea (*μ*mol/L)	Creatinine (*μ*mol/L)	Uric acid (*μ*mol/L)	BUN (mmol/L)	AST (U/L)	ALT (U/L)	LDH (U/L)
Control	35.51 ± 1.57	52.29 ± 0.80	20.39 ± 0.87	4.63 ± 0.22	63.61 ± 1.71	60.12 ± 1.57	21.68 ± 0.98
LEM	35.84 ± 4.51	51.06 ± 1.05	19.37 ± 0.78	4.39 ± 0.64	60.29 ± 2.22	59.28 ± 1.33	20.82 ± 0.37
Cisplatin	68.01 ± 2.03^+++^	96.01 ± 1.80^+++^	30.58 ± 0.25^+++^	8.99 ± 0.14^+++^	111.20 ± 6.47^++^	97.49 ± 2.14^+++^	56.36 ± 1.67^+++^
LEM + cisplatin	39.02 ± 0.65^∗∗∗^	71.98 ± 1.86^∗∗∗^	25.36 ± 0.90^∗∗^	5.66 ± 0.35^∗∗∗^	71.05 ± 1.93^∗∗^	70.43 ± 0.93^∗∗∗^	28.02 ± 1.66^∗∗∗^
Quercetin + cisplatin	40.56 ± 4.34^∗∗^	67.68 ± 1.61^∗∗∗^	22.16 ± 0.96^∗∗^	5.39 ± 0.24^∗∗∗^	65.3 ± 0.86^∗∗^	69.01 ± 2.20^∗∗∗^	22.92 ± 1.70^∗∗∗^

Values represent mean ± SEM from three independent experiments, *n* = 6 mice per group. ^++^Significant difference at *p* < 0.01 and ^+++^significant difference at *p* < 0.001: the cisplatin group versus the control group. ^∗∗^Significant difference at *p* < 0.01 and ^∗∗∗^significant difference at *p* < 0.001: the LEM + cisplatin and quercetin + cisplatin groups versus the cisplatin group.

## Data Availability

All the data supporting the results are shown in the paper and can be available from the corresponding author.
